# Work burnout and community policing among junior-ranked officers of the Nigerian police

**DOI:** 10.3389/fpsyg.2026.1791705

**Published:** 2026-03-11

**Authors:** Happiness Okoka, Jabulani Gilford Kheswa, Sadiq Ewaoda Amali

**Affiliations:** 1 Department of Psychology, University of Fort Hare, Alice, Eastern Cape, South Africa; 2 Department of Criminology and Security Science, University of South Africa, Pretoria, South Africa

**Keywords:** burnout, community policing, depersonalization, emotional exhaustion, Nigeria police, personal accomplishment

## Abstract

This study investigates the psychological impact of burnout on community policing among junior Nigerian police officers. The study employed a quantitative approach using a cross-sectional design, which involved 180 officers from three State Police Commands. Three hypotheses were tested with the Linear Multiple Regression statistical tool. Results showed that Emotional exhaustion significantly negatively predicted the practice of community policing (*β* = −0.289, *t* = −3.196, *p* < 0.05). Personal accomplishment significantly positively predicted the practice of community policing (*β* = 0.387, *t* = 4.865, *p* < 001); and Depersonalization also significantly negatively predicted community policing (*β* = −0.379, *t* = −4.179, *p* < 001). The study suggests that increased emotional exhaustion and depersonalization lead to lower participation in community policing, while higher personal accomplishments encourage more active involvement. The study recommends professional development initiatives, supportive work environments, and comprehensive burnout prevention strategies.

## Introduction

Community policing has emerged as a fundamental approach in modern law enforcement, emphasizing proactive collaboration between the police and the communities they serve (Kappeler et al., 2020). Its primary goal is fostering trust, addressing localized concerns, and creating safe communities. The adoption of community policing in Nigeria reflects efforts to strengthen police-community relationships, enhance public safety, and improve service delivery. However, the effectiveness of these efforts is significantly shaped by the well-being of officers, particularly junior-ranked personnel, who are central to the operationalization of community policing strategies (Golo, 2023).

Burnout among police officers is a prevalent and concerning phenomenon, marked by emotional exhaustion, depersonalization, and a reduced sense of personal accomplishment (Tsirimokou et al., 2024; Queirós et al., 2020). Emotional exhaustion occurs when prolonged stress depletes officers’ emotional reserves, impairing their ability to engage empathetically with community members. Depersonalization manifests as a detached, often cynical attitude toward the public and one’s duties, directly undermining the principles of trust and collaboration central to community policing. Conversely, personal accomplishment—feeling effective and competent in one’s role—has fostered job satisfaction and enhanced community engagement efforts (Ebonwu and Nwankwo, 2023; Maslach and Leiter, 2016).

In the Nigerian context, junior-ranked officers face a unique confluence of stressors, including inadequate resources, exposure to security risks, and conflicting demands between traditional law enforcement and community-oriented policing practices (Bisji et al., 2021). These challenges exacerbate burnout and can diminish officers’ capacity to maintain interpersonal relationships and proactive problem-solving for effective community policing (Bassey, 2023; Akinyemi, 2021). Despite its importance, limited research has systematically explored the relationship between burnout dimensions and the effectiveness of community policing among junior officers in Nigeria, leaving a critical gap in understanding and addressing these issues.

Addressing burnout is essential to ensuring the sustainability of community policing initiatives in Nigeria. Emotional exhaustion may hinder officers’ ability to engage proactively, while depersonalization can lead to strained relationships with community members. A diminished sense of personal accomplishment can further erode officers’ motivation to adopt innovative and collaborative policing strategies. Understanding the interplay between these dimensions of burnout and community policing is critical for designing interventions that support officers’ well-being and enhance their performance (Turgoose et al., 2022; Gumani, 2019).

This study seeks to bridge the existing research gap by examining the influence of emotional exhaustion, depersonalization, and personal accomplishment on community policing practices among junior-ranked Nigerian police officers. By investigating these interconnections, the research aims to offer actionable insights into mitigating burnout and strengthening community policing frameworks. Ultimately, this investigation aligns with broader efforts to enhance policing efficacy through human-centered approaches that prioritize the psychological health and operational effectiveness of police officers. By addressing the multidimensional impacts of burnout, the study will create a resilient police force capable of upholding the principles of trust, partnership, and proactive engagement that underpin community policing in Nigeria and beyond.

## Statement of problem

Community policing in Nigeria relies heavily on junior-ranked officers’ emotional and psychological well-being, as they are central to building relationships with the community and ensuring effective policing strategies. However, burnout—manifested through emotional exhaustion, depersonalization, and a sense of reduced accomplishment—significantly hinders their ability to engage in community-based initiatives effectively. This issue is compounded by high work-related demands, scarce resources, and structural challenges within the police force, all contributing to burnout and impacting officers’ performance and interactions with the public.

Despite the critical role burnout plays in community policing, more research is needed on its impact in the Nigerian context. The existing body of research largely overlooks the psychological toll on officers, focusing instead on broader organizational or external factors. As a result, the connection between burnout and the practice of community policing still needs to be explored. Understanding how burnout affects junior officers’ performance in community policing is essential for developing targeted interventions to support their mental health and improve the overall effectiveness of policing strategies in Nigeria.

## Aim and objectives of the study

The aim of this study is to investigate the role of burnout dimensions (Emotional exhaustion, personal accomplishment, and depersonalization) and the practice of community policing among junior-ranked officers in the Nigerian Police Force. Specific objectives include:

1. To assess the role of emotional exhaustion in predicting the practice of community policing among junior-ranked officers in the Nigerian Police Force.
2. To examine how personal accomplishment predicts the practice of community policing among junior-ranked officers in the Nigerian Police Force.
3. To identify the role of depersonalization in predicting the practice of community policing among junior-ranked officers in the Nigerian Police Force.

## Hypotheses

The following hypotheses were tested in the study.

Emotional exhaustion will significantly negatively predict the effectiveness of community policing among junior-ranked officers in the Nigerian Police Force.

Personal accomplishment will significantly predict junior officers’ engagement in community policing activities.

Depersonalization will significantly negatively predict junior officers’ inability to engage in community policing effectively.

## Method

### Design

The study employed a quantitative approach using a cross-sectional design, which analyses data from a population sample at a specific point in time (Spector, 2019). This design is well-suited for efficiently assessing the prevalence and relationships between variables across different population segments (Olsen and St. George, 2004). By using the cross-sectional approach, the study captures simultaneous data on burnout, and the practice of community policing among junior police personnel, enabling a predictive analysis of these variables and their potential associations. The independent variables in the study include burnout dimensions (emotional exhaustion, personal accomplishment, and depersonalization), while the dependent variable is the practice of community policing.

### Participants

The study included a total of 180 junior officers of the Nigerian police from three State Police Commands. The mean age of the participants was 32.1 years (*SD* = 7), with 122 males and 58 females. A sample size of 129 was determined as the minimum sample size adequate for powering the study using the G-power calculator (Faul et al., 2007) based on a medium effect size of 0.15, a significance level of 0.05, and a power of 0.95. Convenience sampling was employed, and participants who met the inclusion criteria were selected: full-time employees with at least 6 months of service and willingness to participate. This method was justified by the accessibility of eligible participants, enhancing the study’s statistical power (Suen et al., 2014).

### Instruments

Two psychological scales were used to measure the perceptions of the constructs in this study, as behaviour is often driven by individuals’ perceptions of reality, even when their beliefs may be inaccurate (Eisenberger et al., 1990). These scales include the Maslach Burnout Inventory (MBI), which assesses burnout, and the Community Policing Inventory (CPI), which evaluates the practice of community policing.

#### Maslach burnout inventory (MBI)

The Maslach Burnout Inventory (Maslach and Jackson, 1981) is a 22-item tool designed to assess burnout, consisting of three subscales: depersonalization (5 items), emotional exhaustion (9 items), and personal accomplishment (8 items). Each subscale is rated on a five-point Likert scale, ranging from “0” (never) to “5” (always). Emotional exhaustion evaluates feelings of being emotionally drained due to work demands, while depersonalization measures hostile or callous attitudes toward colleagues or the workplace. Personal accomplishment gauges a person’s sense of skill, productivity, and job efficacy. The subscales demonstrate high internal consistency (Cronbach’s alpha values of 0.837 to 0.881) and reliability (Wickremasinghe et al., 2018). High scores in depersonalization and emotional exhaustion and low scores in personal accomplishment indicate higher burnout levels.

#### Community policing inventory (CPI)

The Community Policing Inventory was adapted from the Community Survey on Public Safety and Law Enforcement (CSPLE), developed by the U.S. Department of Justice’s Office of Community Oriented Policing Services (COPS Office) with input from ICF International and law enforcement experts. The inventory consists of 14 items, rated on a five-point Likert scale ranging from 1 (“not at all”) to 5 (“great extent”). Sample items include questions about law enforcement agencies’ relationships with the community and officers’ concern for residents. The scoring range is from 14 to 70, with higher scores indicating greater engagement in community policing. The inventory demonstrated high internal consistency with a Cronbach’s alpha coefficient of 0.901.

### Procedure

The University of Fort Hare’s Research Ethics Committee (REC) granted this study’s ethical approval with reference number ETHICS CLEARANCE REC-270710-028-RA Level 01. Permission to administer questionnaires was obtained from the Nigerian Police Divisional Headquarters in Abuja, Kaduna, and Plateau State after submitting a formal request. The study faced delays in gaining approval due to regional security concerns. During fieldwork, the researcher ensured participants’ anonymity, voluntary participation, and data confidentiality despite challenges such as limited research hours and shift work.

### Method of data analysis

Multiple Linear Regression (MLR) was employed to test the study hypotheses (Equation 1). The MLR is particularly effective in predictive correlational research, allowing the researcher to examine patterns in the relationships between independent (predictor) variables, such as burnout, and the dependent (criterion) variable, the practice of community policing. By applying this statistical approach, the study was able to assess how burnout dimensions predict the practice of community policing among junior police officers.

### Model specification for hypotheses testing

The model for testing the study hypotheses in the research is stated thus:


CP1=βO+β1EExh+β2PAcom+β2Deper+e1 (1)

Where:

*CP*_1_ = Practice of community policing.

*EExh* = Emotional exhaustion.

*PAcom* = Personal accomplishments.

*Deper* = Depersonalisation.

*β*_*O* =_ Intercept.

*β_1_*, *β_2_*, and *β_3_* = Coefficients.

e_1_ = error terms.

Based on *a priori* expectation, it is expected that all the parameter estimates are *β_1_* > 0, μ_1_ < 0, σ_1_ < 0, and α_1_ > 0.

## Results

### Descriptive results

The descriptive results showing the mean, standard deviations, and correlations between the independent and dependent variables, which include perceived self-efficacy, work burnout (Emotional exhaustion, personal accomplishments, and depersonalization), and practice of community policing, are presented in Table 1.

**Table 1:** Mean, standard deviations, and correlations between the independent and dependent variables.

		1	2	3	4
1	Emotional exhaustion	1	0.521^**^	0.664^**^	−0.340^**^
2	Personal accomplishments		1	0.524^**^	0.037
3	Depersonalization			1	−0.369
4	Community policing				1
Mean		13.51	18.46	7.34	54.57
*SD*		7.70	7.49	5.81	10.47

^**^p < 0.01.

Table 1 shows the mean, standard deviation, and correlations between emotional exhaustion, personal accomplishments, depersonalization, and the practice of community policing. The results indicate that emotional exhaustion (*M* = 13.51, *SD* = 7.70) was significantly positively correlated with personal accomplishments (*M* = 18.46, *SD* = 7.49) *r* = 0.521, *p* < 0.01, suggesting a moderate positive relationship between these variables. Emotional exhaustion was also significantly positively correlated with depersonalization (*M* = 7.34, *SD* = 5.81) *r* = 0.664, *p* < 0.01, indicating a strong positive relationship. Additionally, emotional exhaustion showed a significant negative correlation with the practice of community policing (*M* = 54.57, *SD* = 10.47) *r* = −0.340, *p* < 0.01, meaning higher emotional exhaustion is associated with less engagement in community policing. Personal accomplishments (*M* = 18.46, *SD* = 7.49) were positively correlated with depersonalization (*M* = 7.34, *SD* = 5.81) *r* = 0.524, *p* < 0.01, but not significantly associated with community policing (*r* = 0.037, *p* > 0.05). Depersonalisation (*M* = 7.34, *SD* = 5.81) was negatively correlated with community policing (*M* = 54.57, *SD* = 10.47) *r* = −0.369, *p* < 0.01, indicating that higher depersonalization is associated with lower practice of community policing.

### Inferential results

Prior to hypothesis testing, multicollinearity diagnostics were examined to assess the suitability of the predictors for multiple linear regression analysis. The results showed that tolerance values ranged from 0.607 to 0.754, while Variance Inflation Factor (VIF) values ranged from 1.326 to 1.648. These values fall well within the recommended thresholds, indicating that multicollinearity was not a concern and that the regression coefficients were stable and reliable.

Subsequently, three hypotheses were tested using Multiple Linear Regression (MLR) to examine the relationship between work burnout dimensions, emotional exhaustion, personal accomplishment, and depersonalization, and the practice of community policing. The results of the regression analyses are presented below.

Table 2 shows the model summary of the regression for the relationship between work burnout and the practice of community policing. The table indicated that *R*^2^ = 0.253, *F* (3, 176) = 19.841, *p* < 0.001, which indicates that the model accounted for 25.3% of the variance in the practice of community policing.

**Table 2:** Regression model summary for the relationship between work burnout and practice of community policing.

*R* ^2^	Adjusted *R*^2^	Std. error of estimated	*R*^2^ change	*F* change	df1	df2	*p*-value
0.253	0.240	9.128	0.253	19.841	3	176	<0.001

#### Dependent variable: practice of community policing

The results in Table 3 show the coefficients of the multiple linear regression analysis for the relationship between work burnout and the practice of community policing among junior police officers. Emotional exhaustion significantly negatively predicted the practice of community policing (*β* = −0.289, *t* = −3.196, *p* < 0.05), indicating that higher emotional exhaustion is associated with lower engagement in community policing. Personal accomplishment significantly positively predicted the practice of community policing (*β* = 0.387, *t* = 4.865, *p* < 001), suggesting that a greater sense of accomplishment enhances officers’ participation. Depersonalization also significantly negatively predicted community policing (*β* = −0.379, *t* = −4.179, *p* < 001), meaning higher depersonalization leads to lower involvement in community policing.

**Table 3:** Coefficients of the relationship between work burnout and practice of community policing among junior police officers.

	Unstandardized Coefficients		Standardized Coefficients	*t*	*p*-value
	*Β*	Std. error	*β*		
(Constant)	54.929	1.871		29.366	<0.001
Emotional exhaustion	−0.393	0.123	−0.289	−3.196	0.002
Personal accomplishment	0.540	0.111	0.387	4.865	<0.001
Depersonalization	−0.684	0.164	−0.379	−4.179	<0.001

## Discussion of findings

Results indicated that emotional exhaustion significantly negatively predicted the practice of community policing by junior-ranked police officers in Nigeria. This aligns with the findings of Okoka and Kheswa (2024), which found that higher emotional exhaustion correlated with lower practice of community policing. Also, studies have found that emotional exhaustion lowers the practice of community policing (Turgoose et al., 2022; Adams and Mastracci, 2020). The findings that emotional exhaustion significantly negatively predicted the practice of community policing by junior-ranked police officers in Nigeria can be understood within the context of how burnout affects occupational performance. Emotional exhaustion, a key component of burnout, is characterized by feelings of being overextended and depleted of emotional and physical resources (Nápoles, 2022). For police officers who work in a high-stress environment, this exhaustion can lead to decreased motivation, reduced energy levels, and a diminished capacity to engage with community members effectively. As community policing requires proactive engagement, collaboration, and building trust with community members, officers who are emotionally exhausted may lack the psychological and physical energy to carry out these tasks, significantly impacting their ability to practice community policing effectively.

Findings revealed that personal accomplishment significantly positively predicted the practice of community policing by junior-ranked police officers in Nigeria. This suggests that junior officers who experience a sense of achievement and competence in their work are more likely to be effective in community policing roles. This aligns with studies that indicated that personal accomplishment is a protective factor against workplace dissatisfaction (Choi et al., 2024; Torres-Vences et al., 2022; Levin et al., 2022; Bang and Reio, 2017). The outcome could be argued on the premise that personal accomplishment refers to the feelings of efficiency and success that an individual experiences in their job. Police officers who feel competent and successful will likely be more confident, motivated, and engaged. In community policing, these junior officers would be more inclined to take initiative, communicate effectively with community members, and invest effort in building positive relationships with the public, all of which are critical to successfully implementing community policing.

Also, depersonalization was found to significantly negatively predict the practice of community policing by junior-ranked police officers in Nigeria, indicating that officers experiencing higher levels of depersonalization are less likely to adopt and implement community policing principles. This is in agreement with the findings of Tome and van der Vaart (2020), which found a significant association between depersonalization and work performance; also, studies that found a relationship between job satisfaction and depersonalization (Maniscalco et al., 2024; Buys, 2016), as job satisfaction is a predictor of work performance (Baluyos et al., 2019). The finding that depersonalization significantly negatively predicted the practice of community policing highlights the detrimental impact of this aspect of burnout on police work. Depersonalization involves a sense of detachment and a cynical attitude toward one’s job and the people one serves (Larsen et al., 2017). For junior-ranked police officers, high levels of depersonalization could manifest as a lack of empathy and a dismissive attitude toward community members, undermining the core principles of community policing. This approach requires officers to be approachable, empathetic, and actively involved in the community, all of which are hindered when officers experience depersonalization. Consequently, officers who exhibit higher levels of depersonalization are less likely to engage in the collaborative and trust-building activities essential to effective community policing, leading to a significant negative prediction of their practice of community policing.

The findings of this study can be most appropriately situated within the Job Demands–Resources (JD–R) model, which explains how the balance between job demands and available resources shapes employee well-being and performance in high-stress occupations such as policing (Demerouti et al., 2001). Policing is characterized by intense job demands, including emotional labour, exposure to risk, workload pressure, and public scrutiny. When these demands are not adequately offset by job and personal resources, burnout emerges, particularly in the form of emotional exhaustion and depersonalization. In the present study, the significant negative predictive effects of emotional exhaustion and depersonalization on community policing practices suggest that depleted psychological and emotional resources undermine officers’ capacity to engage in the proactive, relational, and trust-based behaviours that community policing requires (Bakker and Demerouti, 2007).

Conversely, personal accomplishment emerged as a positive predictor of community policing, reflecting its role as a vital personal resource within the JD–R framework. Officers who experience a strong sense of competence and achievement are more likely to remain motivated, engaged, and resilient despite demanding work conditions. This enhanced engagement facilitates initiative, effective communication, and sustained collaboration with community members, all of which are central to successful community policing. Taken together, these findings extend the application of the JD–R model to community policing by demonstrating how specific dimensions of burnout shape officers’ capacity to implement community-oriented practices, thereby strengthening the study’s theoretical contribution beyond descriptive and applied insights.

## Implications of the study

The study’s findings have several significant implications for community policing in Nigeria, particularly regarding the well-being and job satisfaction of junior-ranked police officers. The negative impact of emotional exhaustion on the practice of community policing highlights the urgent need for police departments to prioritize the mental health and well-being of their officers. High levels of emotional exhaustion can diminish officers’ motivation, energy, and capacity to engage with community members effectively. To address this, police departments should implement comprehensive stress management programs, provide psychological support, and ensure that officers have manageable workloads. Doing so can reduce emotional exhaustion and enhance officers’ ability to practice community policing, which relies heavily on proactive engagement, collaboration, and trust-building with the public.

Furthermore, the positive relationship between personal accomplishment and community policing suggests that fostering a sense of achievement and competence among officers is crucial for effective community policing. Officers who feel competent and successful are more likely to be motivated, confident, and engaged in their work, translating into more effective community policing practices. Police departments should invest in professional development programs, recognize, and reward officers’ achievements, and create an environment that supports personal and professional growth. By enhancing job satisfaction and feelings of personal accomplishment, departments can improve officers’ commitment to community policing and their ability to build positive relationships with the communities they serve.

Finally, the significant negative prediction of depersonalization on community policing emphasizes the importance of addressing this aspect of burnout among police officers. Depersonalization, which involves a cynical and detached attitude toward the job and the people officers serve, can severely undermine the principles of community policing. To combat this, police departments should build a supportive and empathetic work culture that encourages officers to remain connected and engaged with the community. This may involve providing training on emotional intelligence, promoting peer support systems, and fostering a sense of purpose and meaning in the work that officers do. By addressing depersonalization, police departments can ensure that officers are better equipped to engage in the collaborative and trust-building activities essential to successful community policing.

## Limitations of the study

The study has several limitations that should be acknowledged, as they may influence the interpretation of the findings and the generalizability of the results. Firstly, the study’s reliance on self-reported measures may introduce bias, as participants’ responses could be influenced by social desirability or their current mood. This could lead to either overestimation or underestimation of emotional exhaustion, personal accomplishment, depersonalization, and the practice of community policing. Future research could incorporate objective measures or third-party evaluations to complement self-reported data.

Secondly, the study’s cross-sectional design limits the ability to draw causal inferences from the findings. While the study identifies significant relationships between burnout dimensions and community policing practices, it cannot establish a clear cause-and-effect relationship. Longitudinal studies are needed to track these variables over time and better understand how changes in burnout levels impact community policing behaviour.

Thirdly, the convenience sampling employed poses important limitations for representativeness and external validity. While this approach was practical given access restrictions and the structured nature of policing institutions, it increases the likelihood of selection bias. Officers who were more accessible or willing to participate may systematically differ from those who were not included, particularly in terms of workload, stress levels, or attitudes toward community policing. As a result, the findings may not fully reflect the experiences of the wider police population, and caution is required when generalizing the results beyond the sampled officers. Future research should consider probability-based sampling techniques, where feasible, or multi-site sampling to enhance representativeness and strengthen the generalizability of findings.

Another area for improvement is the focus on junior-ranked police officers, which may restrict the generalizability of the findings to other ranks within the police force. Senior officers, for example, may experience burnout differently and have different influences on their community policing practices. Expanding the study to include a broader range of police ranks would provide a more comprehensive understanding of how burnout affects community policing across the force. Additionally, the study was conducted within a specific cultural and organizational context, Nigeria’s police force, which may limit the generalizability of the findings to other countries or policing environments. Cultural differences in policing practices, community relations, and organizational structures could lead to different outcomes in other settings. Future research should consider replicating the study in different cultural and organizational contexts to explore the universality of the findings.

Despite these limitations, the findings of the study remain valuable and meaningful. The study provides important empirical insights into the relationship between burnout dimensions and community policing practices among police officers within a challenging and under-researched context. The study contributes to the growing body of literature on occupational stress and policing in developing countries. Furthermore, the findings offer a useful evidence base for policy formulation and intervention planning aimed at improving officer well-being and strengthening community-oriented policing practices.

## Conclusion

The study concludes that the dimensions of burnout—emotional exhaustion, personal accomplishment, and depersonalization—significantly influence the practice of community policing among junior-ranked police officers in Nigeria. Specifically, emotional exhaustion and depersonalization were found to negatively impact officers’ ability to engage in community policing, while a sense of personal accomplishment positively predicted effective community policing practices. These findings underscore the critical role of psychological well-being and job satisfaction in enabling officers to perform their duties effectively within a community-oriented policing framework.

The study highlights the need for police departments to prioritize their officers’ mental health and job satisfaction to enhance the effectiveness of community policing initiatives. Addressing burnout through comprehensive support systems, professional development, and a positive organizational culture can improve officers’ engagement with the community and overall performance. Moreover, the study’s insights suggest that fostering a sense of achievement and competence among officers is essential for the success of community policing efforts. While the study provides valuable insights, it also acknowledges limitations, such as the reliance on self-reported data and the cross-sectional design, which may affect the generalizability and interpretation of the findings. Future research should explore these relationships further, considering longitudinal approaches and broader samples to enhance understanding and application across.

## Recommendations

Based on the outcomes of the study, several recommendations can be made to enhance the practice of community policing among junior-ranked police officers in Nigeria:

1. Implement Stress Management and Support Programs: To mitigate emotional exhaustion, police departments should develop and implement comprehensive stress management and psychological support programs. These programs could include regular mental health screenings, counselling, and stress reduction workshops. By addressing emotional exhaustion proactively, departments can help officers maintain their well-being and improve their capacity to engage effectively in community policing.
2. Promote Professional Development and Recognition: Given the positive impact of personal accomplishment on community policing, it is essential to invest in professional development opportunities for officers. This could involve providing training programs and career advancement opportunities and recognizing and rewarding officers for their achievements and contributions. Enhancing officers’ sense of competence and success will likely increase their motivation and effectiveness in community policing.
3. Foster a Supportive Work Environment: To counteract depersonalization and its negative effects, police departments should create a supportive and empathetic work environment. This can be achieved by promoting team-building activities, encouraging open communication, and providing peer support. A positive work culture can help officers maintain a sense of connection and empathy, which is crucial for effective community policing.
4. Develop Comprehensive Burnout Prevention Strategies: Given the complex nature of burnout, police departments need to adopt a holistic approach to prevention and management. This includes regular assessment of burnout levels among officers, addressing workload issues, and ensuring that officers have access to resources that support their mental and emotional health. Prevention strategies should be tailored to junior-ranked officers’ specific needs and challenges.

## Data Availability

The raw data supporting the conclusions of this article will be made available by the authors, without undue reservation.
